# MOV10 Helicase Interacts with Coronavirus Nucleocapsid Protein and Has Antiviral Activity

**DOI:** 10.1128/mBio.01316-21

**Published:** 2021-09-14

**Authors:** Li Wang, Isabel Sola, Luis Enjuanes, Sonia Zuñiga

**Affiliations:** a Department of Molecular and Cell Biology, National Center of Biotechnology, Universidad Autónoma de Madrid, Madrid, Spain; Fred Hutchinson Cancer Research Center; University of Washington

**Keywords:** antiviral response, coronavirus, highly pathogenic coronavirus, nucleocapsid protein, virus-host interactions

## Abstract

Coronaviruses (CoVs) are emergent pathogens that may cause life-threatening respiratory diseases in humans. Understanding of CoV-host interactions may help to identify novel therapeutic targets. MOV10 is an RNA helicase involved in different steps of cellular RNA metabolism. Both MOV10 antiviral and proviral activities have been described in a limited number of viruses, but this protein has not been previously associated with CoVs. We found that during Middle East respiratory syndrome coronavirus (MERS-CoV) infection, MOV10 aggregated in cytoplasmic structures colocalizing with viral nucleocapsid (N) protein. MOV10-N interaction was confirmed by endogenous MOV10 coimmunoprecipitation, and the presence of other cellular proteins was also detected in MOV10 complexes. MOV10 silencing significantly increased both N protein accumulation and virus titer, with no changes in the accumulation of viral RNAs. Moreover, MOV10 overexpression caused a 10-fold decrease in viral titers. These data indicated that MOV10 has antiviral activity during MERS-CoV infection. We postulated that this activity could be mediated by viral RNA sequestration, and in fact, RNA immunoprecipitation data showed the presence of viral RNAs in the MOV10 cytoplasmic complexes. Expression of wild-type MOV10 or of a MOV10 mutant without helicase activity in MOV10 knockout cell lines, developed by CRISPR-Cas technology, indicated that the helicase activity of MOV10 was required for its antiviral effect. Interestingly MOV10-N interaction was conserved in other mildly or highly pathogenic human CoVs, including the recently emerged severe acute respiratory syndrome coronavirus 2 (SARS-CoV-2), although MOV10 antiviral activity was found only in highly pathogenic CoVs, suggesting a potential role of MOV10 in the modulation of human CoVs pathogenesis.

## INTRODUCTION

Middle East respiratory syndrome coronavirus (MERS-CoV) is a life-threatening human virus that emerged during the summer of 2012 in Saudi Arabia (1). As of 28 February 2021, a total of 2,566 cases have been confirmed, distributed over 27 countries, causing 882 deaths (fatality rate, ∼35%) (http://www.who.int/emergencies/mers-cov/en/). The high case fatality rate, continuous MERS-CoV outbreaks, and risk of virus adaptation potentially resulting in pandemic spread make research on MERS-CoV an international priority (2, 3). Furthermore, a novel human CoV, severe acute respiratory syndrome coronavirus 2 (SARS-CoV-2), emerged in December 2019 and has caused a global epidemic with more than 192 million cases and 4.1 million deaths worldwide (21 July 2021) (4, 5). The emergence of SARS-CoV-2 has highlighted the need for flexible, broad therapeutic strategies to fight current circulating CoVs and to control future epidemics. Analysis of the host cell pathways modified by the infection is required to understand CoV pathogenesis, as it may provide novel targets for antiviral strategies.

MERS-CoV is classified within the subgenus *Merbecovirus* of genus *Betacoronavirus* (6, 7). The positive-sense single-stranded RNA genome of MERS-CoV is approximately 30 kb and contains 11 open reading frames (ORFs) in the order 5′-ORF1a-ORF1b-S-3-4a-4b-5-E-M-N-8b-3′ which are expressed from a nested set of eight mRNAs (8). The genus-specific genes 3, 4a, 4b, and 5 are nonessential for virus replication (9, 10). In overexpression analyses, these genes have been shown to be involved in the modulation of virus-host interaction (11, 12), although there is limited information on the role of genus-specific genes in the context of viral infection (10, 13–16). CoV nucleocapsid (N) protein is a multifunctional phosphoprotein essential for the CoV life cycle, with relevant structural and functional roles in viral RNA synthesis (17). In addition, CoV N protein is an essential factor for virus cycle, affecting multiple pathways in the infected cell. N protein is involved in deregulation of the host cell cycle, antagonizes interferon (IFN) production, upregulates the activity of transcription factors involved in inflammation, induces apoptosis, inhibits translation, and interacts with many cellular partners (18–20). Nevertheless, in general, the molecular mechanisms by which CoV N protein interacts with the host cell and influences virus pathogenesis remain to be determined. This is mainly due to the essential and multifunctional characteristics of N protein that make its modification, in the context of virus infection, a challenge.

Cytoplasmic RNA granules are dynamic cellular structures that play essential roles in cell growth and development and in immune and stress responses (21, 22). These cellular foci have specific functions in RNA metabolism, such as in transcription, modification, processing, decay, translation, and arrest. They form a large regulatory network in eukaryotic cells, mediated by communication between different RNA granule types and by sharing protein components (23, 24). Viruses interact with these RNA granules, which may have a proviral or an antiviral effect (25–28). MOV10 protein has been associated with stress granules (SGs) and P bodies (29–31), although in human cells, most of the endogenous MOV10 localizes in the nucleus (32), whereas stress granules are located in the cytoplasm. MOV10 is an RNA helicase of the DExD superfamily and the UPF1-like group of helicases (29, 33). This protein has more than 1,060 interactors, according to the BioGRID database (34), some of them linking MOV10 helicase to other RNA metabolism pathways, such as the nonsense-mediated decay (NMD) pathway or the microRNA (miRNA) or small interfering RNA (siRNA) gene silencing pathways. In addition, *MOV10* is an interferon-stimulated gene (ISG) (35), and MOV10 protein is involved in IFN induction after viral infection (36).

Both proviral and antiviral functions have been reported for MOV10. It is required for hepatitis delta virus (HDV) replication but not for the translation of its mRNA (37), facilitates enterovirus replication (38), inhibits replication of HIV-1 and other retroviruses at multiple steps (39), inhibits nuclear import of influenza virus nucleoprotein (40), inhibits porcine reproductive and respiratory syndrome virus (PRRSV) replication by avoiding nucleocapsid protein trafficking to the nucleus (41), inhibits hepatitis C virus (HCV) and dengue virus replication by partly unknown mechanisms (42, 43), and inhibits bunyavirus replication by blocking several nucleoprotein functions (44). In the case of hepatitis B virus (HBV), contradictory data have been reported for both its proviral (45) and antiviral (46) activities.

In this work, we report that MOV10 interacts with MERS-CoV N protein and that it has antiviral activity, most likely mediated by viral RNA sequestration in cytoplasmic ribonucleoprotein complexes. Interestingly, although the binding between N and MOV10 was conserved in all the human CoVs tested, MOV10 antiviral activity was also observed for pathogenic SARS-CoV-2 but not for mildly pathogenic HCoV-229E. The data presented here reveal a novel complex network of interactions between viral and cellular RNAs and proteins modulating antiviral responses against CoVs.

## RESULTS

### Analysis of MOV10 interaction with MERS-CoV N protein.

**FIG 1:**
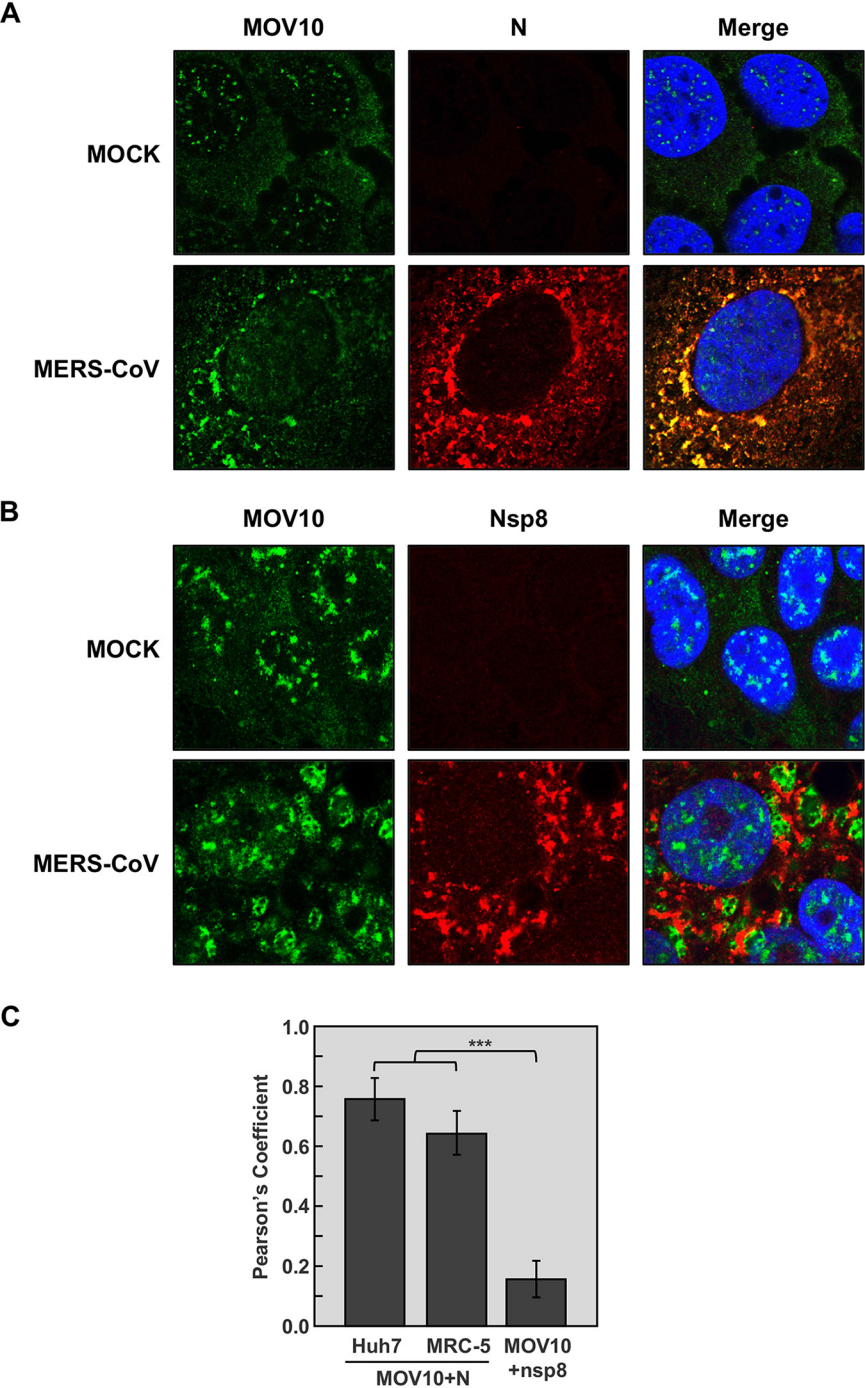
Colocalization of MOV10 and N protein in MERS-CoV-infected cells. Confocal immunomicroscopy analysis was performed on Huh-7 cells that were mock infected or infected with MERS-CoV at an MOI of 0.1. At 20 hpi, cells were fixed and stained with antibodies specific for MOV10 (green; left) and either (A) MERS-CoV N protein (red; middle) or (B) CoV nsp8 protein (red; middle). Cell nuclei, stained with Hoechst 33342 (blue; right), are shown in merged layers. (C) Pearson’s correlation coefficients. The data represent the medians from 20 cells in two independent experiments. Error bars represent SDs. ***, *P* < 0.001.

**FIG 2:**
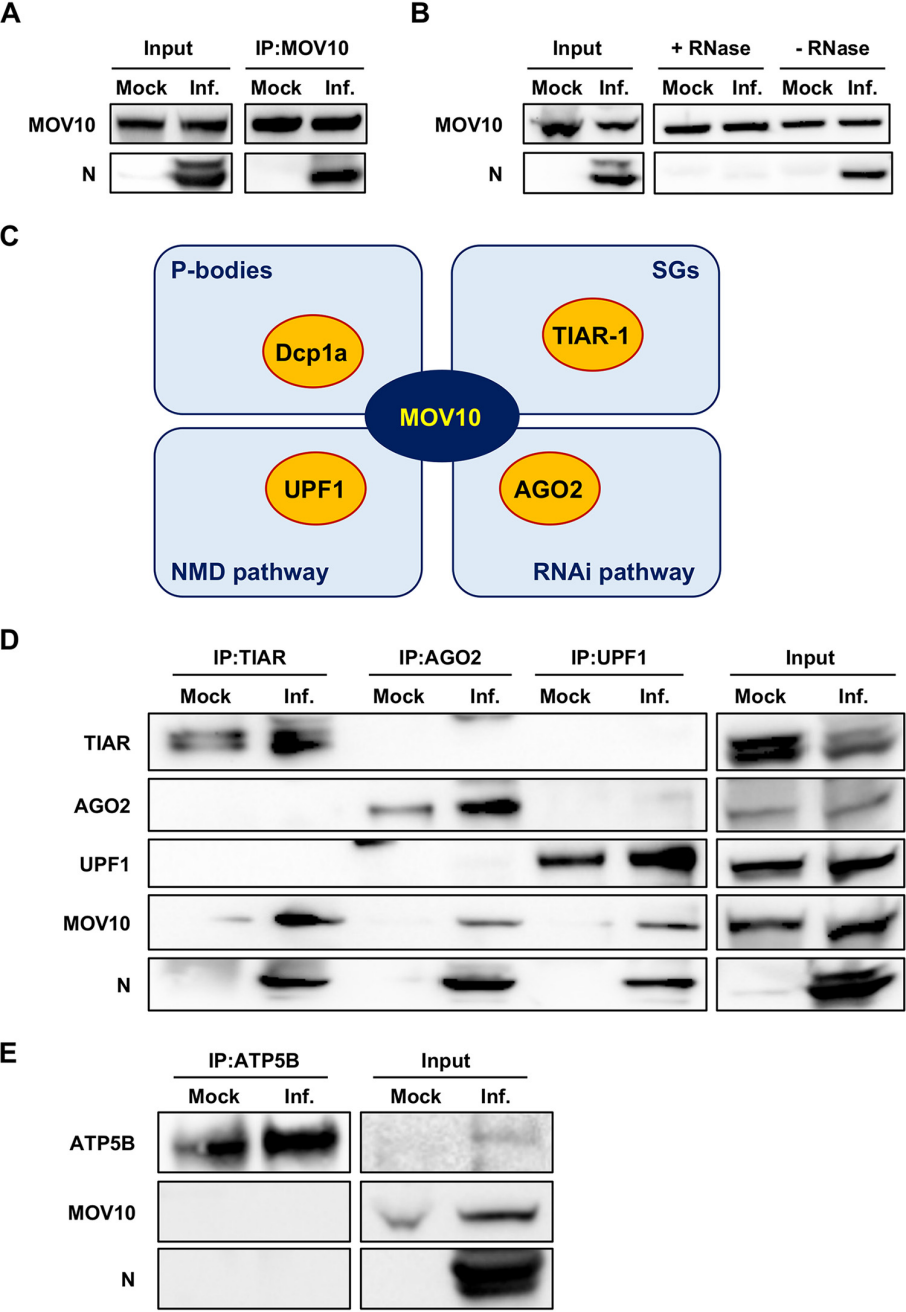
MERS-CoV N protein interacts with MOV10 and related cellular partners. Huh-7 cells were mock infected or infected with MERS-CoV at an MOI of 0.1 (Inf.) for 20 h. (A) Cell lysates were immunoprecipitated with anti-MOV10 antibody. (B) Cell lysates were immunoprecipitated with anti-MOV10 antibody with or without RNase treatment. (C) Schematic diagram of the interaction between MOV10 and selected cellular partners (yellow) involved in different RNA metabolism pathways (light blue). (D) Cell lysates were immunoprecipitated with anti-TIAR, anti-AGO2, or anti-UPF1 antibodies. (E) Cell lysates were immunoprecipitated with anti-ATP5B antibody. In all cases, protein presence in the original extract (input) or in the immunoprecipitated samples (IP) was detected by Western blotting using specific antibodies.

RNA helicase MOV10 was previously identified by our group as a cellular protein included in highly purified transmissible gastroenteritis virus (TGEV) virions, and the interaction of TGEV N protein with MOV10 was observed (A. Nogales, F. Almazan, and L. Enjuanes, unpublished results). Due to the relevant functions of MOV10, we analyzed whether the interaction between MOV10 and N protein also occurs during MERS-CoV infection. Immunofluorescence analysis showed that MOV10 was mainly distributed in the cytoplasm and also in the nucleus of both Huh-7 (Fig. 1A) and MRC-5 (Fig. S1) mock-infected cells. Interestingly, MOV10 subcellular localization changed in MERS-CoV-infected cells, forming cytoplasmic granules both in Huh-7 (Fig. 1A) and MRC-5 (Fig. S1) cells. Moreover, MOV10 extensively colocalized with MERS-CoV N protein in the cytoplasm, mainly in the larger granules (Fig. 1A and C and Fig. S1). The subcellular pattern was reminiscent of that for CoV replication-transcription complexes (RTCs) (47). No colocalization was detected between MOV10 and CoV replicase proteins components of viral RTCs, such as nsp8 (Fig. 1B and C) or nsp14 (data not shown). These data strongly suggested an interaction between MOV10 and MERS-CoV N protein in cytoplasmic ribonucleoprotein structures not related to viral RTCs. The interaction between MOV10 and N was further confirmed by coimmunoprecipitation (co-IP) analysis, as N protein was pulled down together with MOV10 during MERS-CoV infection (Fig. 2A). The interaction of N protein with MOV10 was mediated by RNA, since RNase A treatment eliminated the N protein presence in pulled-down MOV10 complexes (Fig. 2B). This result was in line with other described MOV10 interactions that were also RNA dependent (43, 44, 48, 49). These data demonstrated that MOV10 interacted with MERS-CoV N protein in an RNA-dependent manner.

10.1128/mBio.01316-21.1 FIG S1 Colocalization of MOV10 and MERS-CoV N protein. Confocal immunomicroscopy analysis was performed on MRC-5 cells infected with MERS-CoV at an MOI of 0.1 for 20 h. Colocalization analysis of MOV10 (green; left) and N protein (red; middle) in mock-infected or MERS-CoV-infected cells. Cell nuclei, stained with Hoechst 33342 (blue; right), are shown in merged layers. Download FIG S1, TIF file, 1.5 MB. Copyright © 2021 Wang et al. 2021 Wang et al. https://creativecommons.org/licenses/by/4.0/ This content is distributed under the terms of the Creative Commons Attribution 4.0 International license.

There are more than 1,000 interactors for MOV10 in the BioGRID database (34). Interestingly, some of them belong to cytoplasmic ribonucleoprotein complexes (RNPs) involved in antiviral activity (Fig. 2C), such as Dcp1a (P bodies), AGO2 (RNA interference [RNAi] pathway), and UPF1 (NMD pathway) (30). In addition, MOV10 has been detected as a component of SGs (31). Since TIAR is one of the main nucleation components for these RNPs (31), we chose TIAR for subsequent analyses. The interaction of MOV10 partners with MERS-CoV N protein was analyzed by immunoprecipitation. Endogenous MOV10 protein interaction in mock-infected cells was detected only for TIAR (Fig. 2D). In contrast, both MOV10 and N protein were pulled down together with TIAR, AGO2, or UPF1 proteins in infected cells (Fig. 2D). It is worth noting that, since the blots shown in Fig. 2D are from a unique membrane, there was no simultaneous detection of TIAR, AGO2, or UPF1 when pulldown was performed with antibodies specific for any of these proteins. These data strongly suggested that MOV10 and N interaction with these cellular proteins is not simultaneous. Unfortunately, interaction between Dcp1a, MOV10, and N protein could not be analyzed, as the available anti-Dcp1a antibody did not work in immunoprecipitation. To rule out MOV10 being a sticky protein pulled down nonspecifically in infected cells, ATP synthase subunit beta (ATP5B) was immunoprecipitated as a negative control. Neither MOV10 or N was pulled down with ATP5B (Fig. 2E), indicating that TIAR, AGO2, and UPF1 are specific interacting partners. Together, the results indicated that cytoplasmic structures containing MOV10 and MERS-CoV N protein may potentially include other proteins, such as TIAR, AGO2, or UPF1, involved in antiviral functions.

### Effect of MERS-CoV infection on MOV10 levels.

To analyze the effect of MERS-CoV infection on MOV10 expression, Huh-7 cells were infected with MERS-CoV at a MOI of 1 and samples were collected at different times postinfection. No significant changes in MOV10 mRNA accumulation during MERS-CoV infection were observed compared with mock-infected cells, as determined by reverse transcription-quantitative PCR (RT-qPCR) (Fig. S2A). Accordingly, no significant difference was observed in MOV10 protein accumulation during MERS-CoV infection, in either cytoplasmic or nuclear fractions (Fig. S2B). It is worth noting that a limited amount of N protein was detected in the nuclear fraction (Fig. S2B), indicating that MERS-CoV N protein was located in the nucleus at some stages of infection.

10.1128/mBio.01316-21.2 FIG S2 Effect of MERS-CoV infection on MOV10 levels. Huh-7 cells were infected with MERS-CoV at an MOI of 1 for 8, 18, and 24 hpi. (A) Viral gRNA (blue), sgmRNA-N (red), and MOV10 mRNA (gray) accumulation were analyzed by RT-qPCR. The values are means from three independent infections; error bars represent SD. (B) Cytoplasmic and nuclear fractions were analyzed by Western blotting for MOV10 and N protein accumulation. GAPDH and histone H3 were used as cytoplasmic and nuclear markers, respectively. Download FIG S2, TIF file, 0.5 MB. Copyright © 2021 Wang et al. 2021 Wang et al. https://creativecommons.org/licenses/by/4.0/ This content is distributed under the terms of the Creative Commons Attribution 4.0 International license.

### MOV10 activity on MERS-CoV infection.

**FIG 3:**
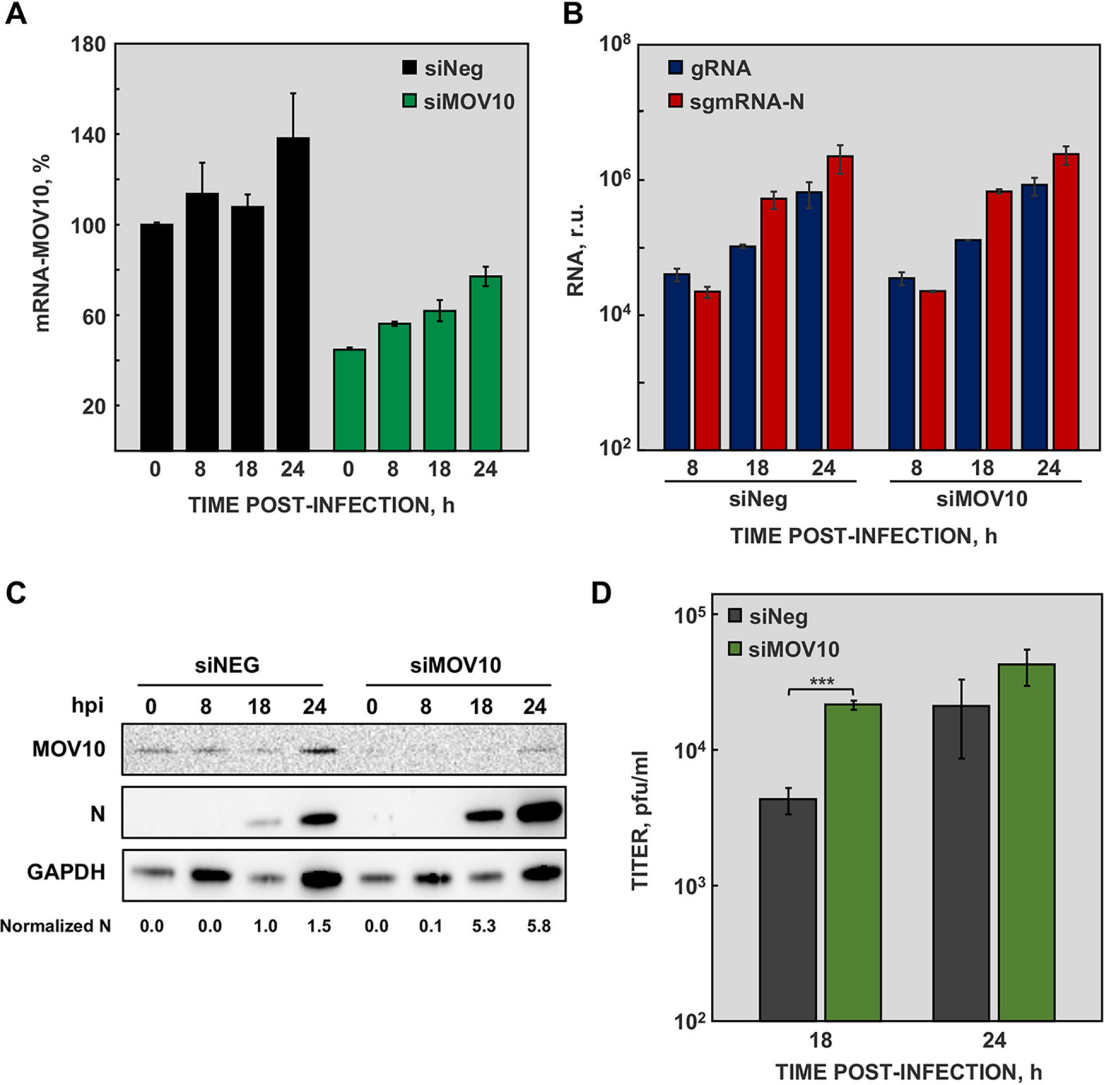
MOV10 activity on MERS-CoV infected cells. (A) Huh-7 cells were transfected with negative-control siRNA (siNeg; black) or MOV10-specific siRNAs (siMOV10; green), and the accumulation of MOV10 mRNA was quantified by RT-qPCR at the indicated time points. (B) Viral gRNA (blue) and sgmRNA-N (red) accumulation, at the indicated time points, was quantified by RT-qPCR in cells transfected with siMOV10 or with siNeg. (C) Cytoplasmic extracts were obtained from cells transfected with siNEG or siMOV10 and mock infected (0) or infected with MERS-CoV at an MOI of 1 for 8, 18, and 24 hpi. MOV10 and MERS-CoV N protein were detected by Western blotting. GAPDH was used as a loading control. Numbers under the blots indicate the estimated levels of N protein, normalized to GAPDH levels and relative to nonsilenced cells at 18 hpi. (D) Virus titers obtained from Huh-7 cells transfected with siNeg (gray) or siMOV10 (green) and infected with MERS-CoV. The values are means from three independent infections; error bars represent SD. ***, *P* < 0.001.

To explore the functional relevance of MOV10 during MERS-CoV infection, its expression was silenced in Huh-7 cells, using a specific siRNA. Silencing caused up to 60% reduction in MOV10 mRNA accumulation (Fig. 3A), although silencing was not stably maintained during the experiment time course (Fig. 3A).

**FIG 4:**
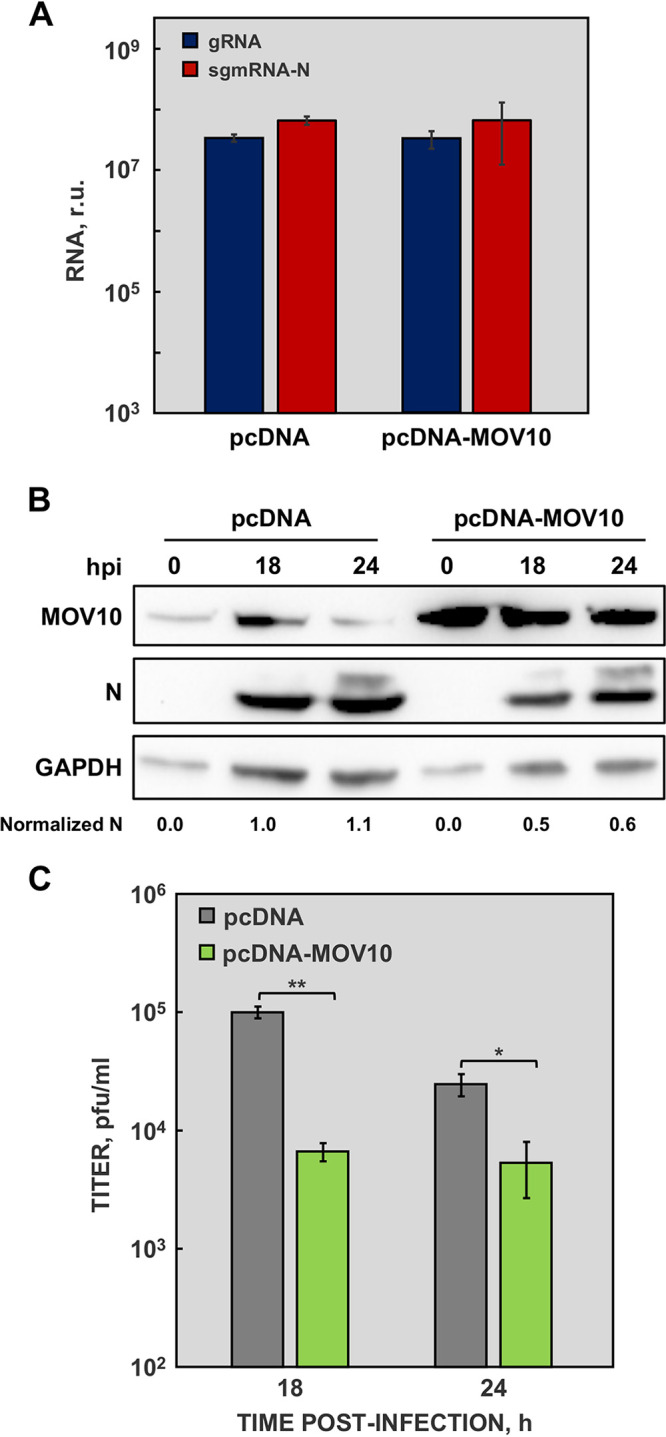
MOV10 activity on MERS-CoV infected cells overexpressing MOV10. (A) Huh-7 cells were transfected with an empty vector (pcDNA) or with a plasmid expressing MOV10 (pcDNA-MOV10) and infected with MERS-CoV at an MOI of 1. Viral gRNA (blue) and sgmRNA-N (red) were quantified by RT-qPCR at 24 hpi. (B) Cytoplasmic extracts were obtained from cells transfected with an empty vector (pcDNA) or with a plasmid expressing MOV10 (pcDNA-MOV10) and infected with MERS-CoV at a MOI of 1 for 18 and 24 hpi. MOV10 and MERS-CoV N protein were detected by Western blotting. GAPDH was used as a loading control. Numbers under the blots indicate the estimated levels of N protein, normalized to GAPDH levels and relative to pcDNA transfected cells at 18 hpi. (C) Virus titers obtained from Huh-7 cells transfected with an empty vector (pcDNA; gray) or with a plasmid expressing MOV10 (pcDNA-MOV10; green) and infected with MERS-CoV. The values are means from three independent infections; error bars represent SD. *, *P* < 0.05; **, *P* < 0.01.

At early times postinfection, N protein accumulates in perinuclear cytoplasmic granules corresponding to RTCs (47). As indicated above, the cytoplasmic structures observed in MERS-CoV-infected cells resemble CoV RTCs, although no colocalization with replicase nonstructural proteins (nsps) was detected (Fig. 1B and C). The effect of MOV10 on viral RNA accumulation was analyzed by RT-qPCR. No significant differences in viral RNA, in either genomic RNA (gRNA) or subgenomic mRNA (sgmRNA) accumulation, were detected in MOV10-silenced cells (Fig. 3B), compared with nonsilenced cells. In line with these data, MOV10 overexpression did not affect viral RNA accumulation (Fig. 4A). These results indicated that MOV10 had no relevant function in viral RNA synthesis.

In contrast, MERS-CoV N protein accumulation was significantly increased in MOV10-silenced cells (Fig. 3C). Moreover, a 5-fold increase in MERS-CoV titers was observed in silenced cells, compared with nonsilenced cells (Fig. 3D). On the other hand, MOV10 overexpression caused a decrease in MERS-CoV N protein accumulation compared with nonoverexpressing cells (Fig. 4B). In agreement with these data, MERS-CoV titers were up to 15-fold lower in cells overexpressing MOV10 (Fig. 4C). Taken together, these data demonstrated that MOV10 has antiviral activity during MERS-CoV infection.

### Antiviral activity of MOV10 occurs posttranscriptionally.

**FIG 5:**
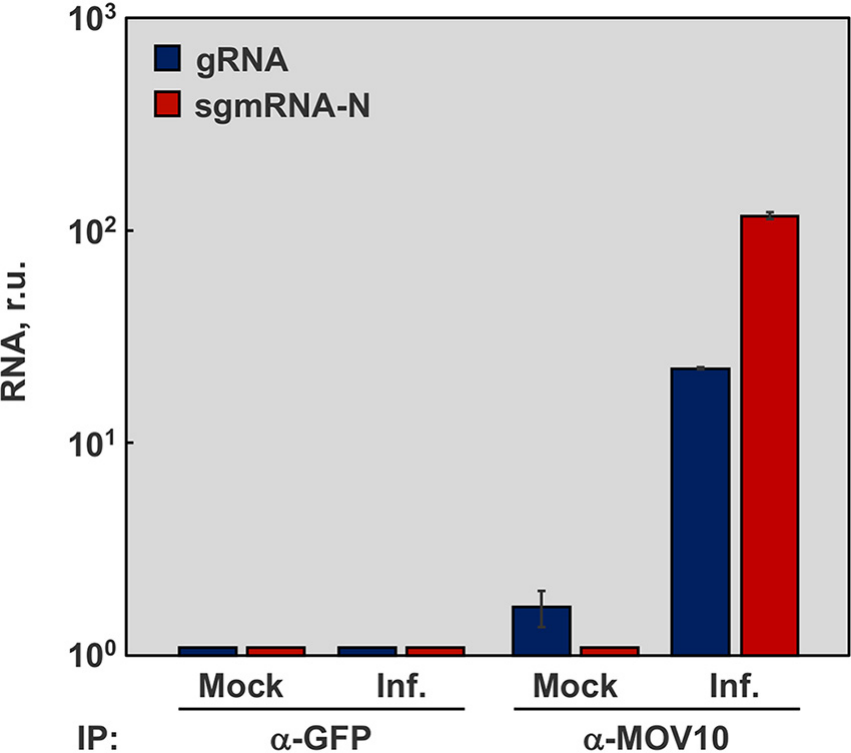
RNA-IP of MOV10 complexes. Huh-7 cells were either mock infected or infected (Inf.) with MERS-CoV at a MOI of 0.1. At 20 hpi, RNA-protein complexes were immunoprecipitated with anti-MOV10 or negative-control anti-GFP antibodies. RNAs eluted from immunoprecipitated RNA-protein complexes were analyzed by RT-qPCR to quantify the associated viral gRNA (blue) and mRNA N (red). The values are means from three independent infections; error bars represent SD.

MERS-CoV viral RNAs accumulation did not change in the absence of MOV10, but viral protein levels and viral titers were markedly increased, suggesting that MOV10 acts in posttranscriptional regulation of viral gene expression to exert its antiviral activity. To further explore this observation, RNA immunoprecipitation (RNA-IP) assays were performed to analyze whether viral RNAs were present in the MOV10 complexes. Extracts from MERS-CoV infected Huh-7 cells were collected and immunoprecipitated with anti-MOV10 antibody, and the presence of RNA in the complexes was quantified by RT-qPCR. A significant 20-fold increase in viral gRNA was observed in MOV10 complexes compared with the negative control immunoprecipitated with an irrelevant antibody (anti-green fluorescent protein [GFP]) (Fig. 5). Similarly, viral mRNA-N was significantly increased 100-fold in MOV10 complexes (Fig. 5). This observation was in line with the recent description of MOV10 as a protein binding CoV RNA (50, 51). Together, the data suggested that MOV10 antiviral mechanism is associated with MERS-CoV RNA sequestration in cytoplasmic granules.

### Effect of MOV10 absence on the interaction with N protein and related cellular partners.

**FIG 6:**
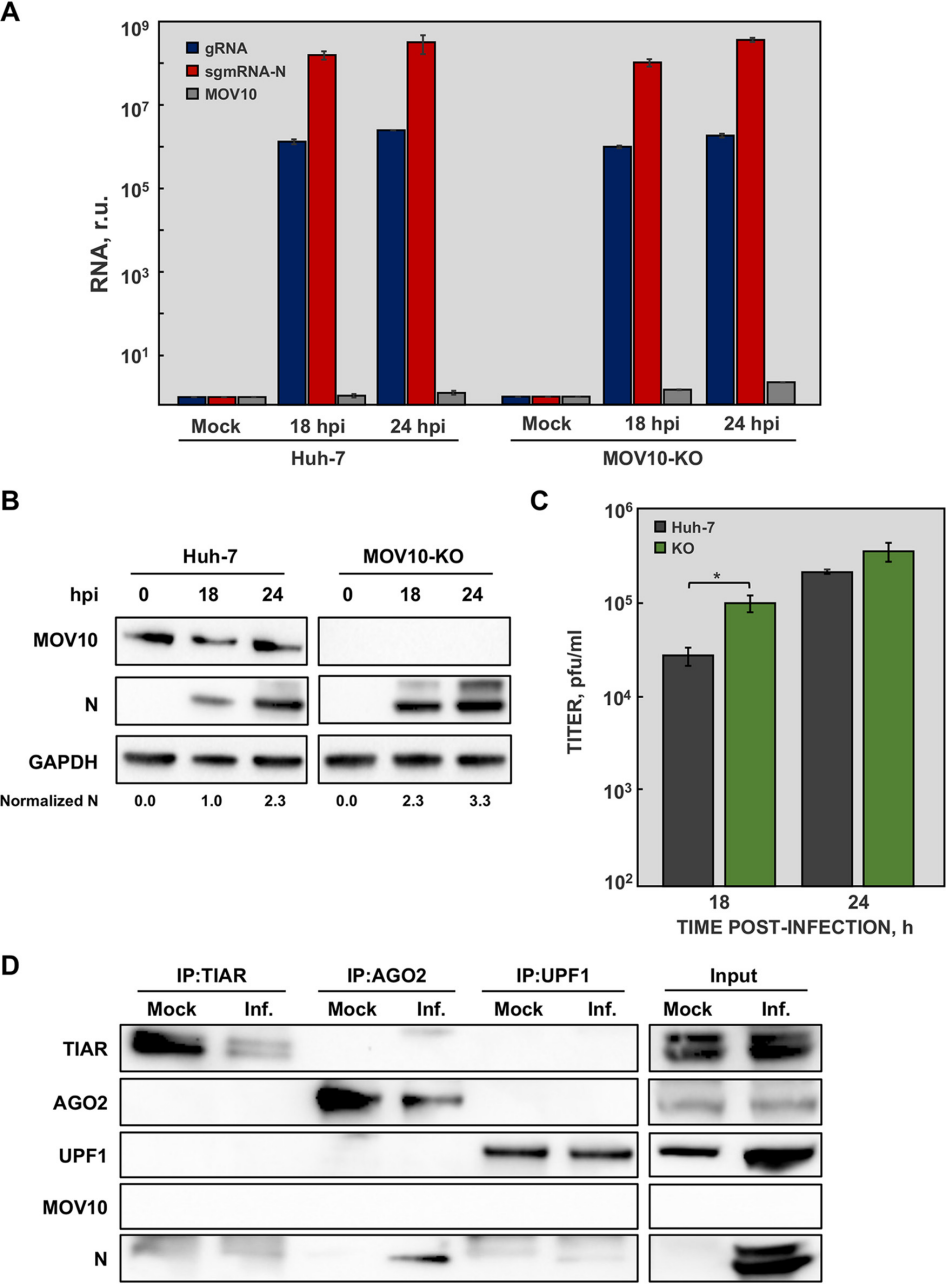
Effect of MOV10 absence on N protein interaction with related cellular partners. Huh-7 and MOV10-KO cells were mock infected or infected with MERS-CoV at a MOI of 1. (A) At the indicated time points, viral gRNA (blue), mRNA N (red), and MOV10 mRNA (gray) accumulation was analyzed by RT-qPCR. (B) In addition, cytoplasmic extracts were analyzed by Western blotting for detection of MOV10 and N protein. GAPDH was used as a loading control. Numbers under the blots indicate the estimated levels of N protein, normalized to GAPDH levels and relative to Huh-7 cells at 18 hpi. (C) Virus titers obtained in Huh-7 (blue) and MOV10-KO (red) cells infected with MERS-CoV. The values are means from three independent infections; error bars represent SD. *, *P* < 0.05. (D) MOV10-KO cells were mock infected or infected with MERS-CoV (Inf.) at an MOI of 0.1. At 20 hpi, cell lysates were obtained and immunoprecipitated with anti-TIAR, anti-AGO2, or anti-UPF1 antibodies. Proteins in the original extract (input) or in the immunoprecipitated samples (IP) were detected by Western blotting using specific antibodies.

MOV10 is involved in the RNAi pathway (29, 36, 52), meaning that MOV10 silencing could be difficult. Thus, it was possible that the previous results obtained during MOV10 silencing were affected by this issue. To exclude this possibility and to analyze the effect of complete MOV10 absence in MERS-CoV infection, a Huh-7 knockout cell line for MOV10 (MOV10-KO) was obtained using CRISPR-Cas9 technology. The replication kinetics of MERS-CoV in MOV10-KO cells was significantly different from that in conventional Huh-7 cells, with a 4-fold increase in maximum viral titers (Fig. S3A). These data confirmed that MOV10 promoted virus replication in cell culture. As expected, the absence of MOV10 did not affect viral RNA accumulation (Fig. 6A), confirming that MOV10 had no relevant role in viral RNA synthesis. Interestingly, both MERS-CoV N protein accumulation (Fig. 6B) and viral titers (Fig. 6C) were significantly increased in MOV10-KO cells, confirming the results previously obtained by MOV10 silencing and reinforcing the antiviral activity of MOV10 during MERS-CoV infection.

10.1128/mBio.01316-21.3 FIG S3 Growth kinetics of human coronaviruses in MOV10-KO cells. Huh-7 or MOV10-KO cells were infected at an MOI of 0.1 with MERS-CoV (A), SARS-CoV-2 (B), or HCoV-229E (C). Supernatants were collected at 24, 48, and 72 hpi and titrated by plaque assay. The values are means from three independent infections; error bars represent SD. *, *P* < 0.05. Download FIG S3, TIF file, 0.4 MB. Copyright © 2021 Wang et al. 2021 Wang et al. https://creativecommons.org/licenses/by/4.0/ This content is distributed under the terms of the Creative Commons Attribution 4.0 International license.

Several host factors interacted with MOV10 and MERS-CoV N protein as described above (Fig. 2D). To investigate whether the interaction between MOV10 partners and MERS-CoV N protein was mediated by MOV10, co-IP analysis was performed in MOV10-KO cells. When TIAR and UPF1 were immunoprecipitated in MOV10-KO cells, the amount of pulled-down N protein was decreased (Fig. 6D), compared to that observed in the presence of MOV10 (Fig. 2D). MERS-CoV N protein was detected in AGO2 complexes (Fig. 6D), although its level was reduced compared with those observed in the presence of MOV10 (Fig. 2D). These results suggested that MOV10 acted as a hub for MERS-CoV N protein interaction with cell proteins involved in antiviral pathways.

### MOV10 helicase activity is required for its antiviral function.

**FIG 7:**
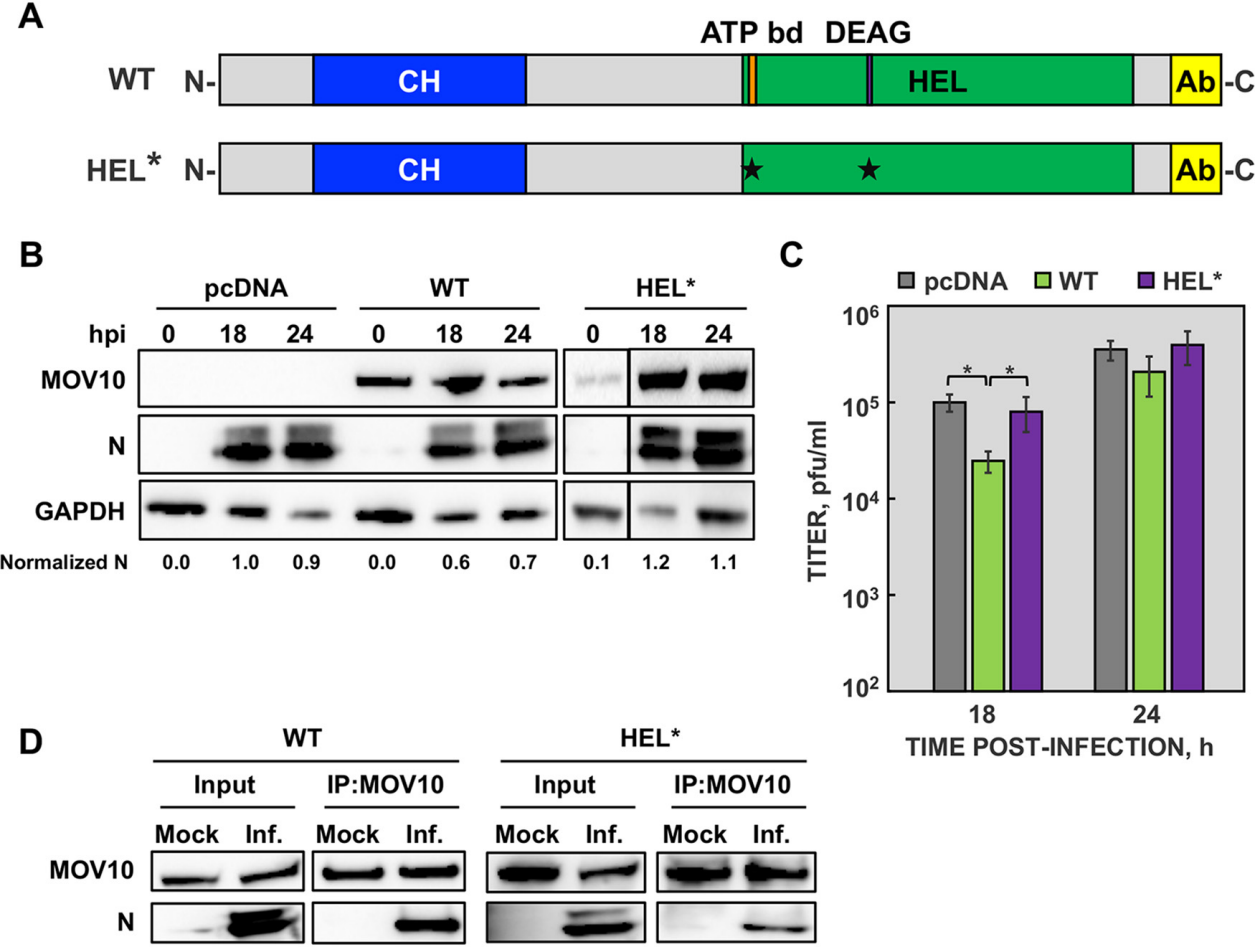
MOV10 helicase activity is required for its antiviral function. (A) Schematic representation of wild-type (WT) and helicase mutant (HEL*) MOV10 proteins. CH, CH-rich domain; HEL, helicase domain; ATP bd, ATP binding motif; DEAG, conserved DEAG box; Ab, domain recognized by MOV10 antibody. The asterisks indicate the point mutations introduced. (B) MOV10-KO cells were transfected with an empty plasmid or plasmids expressing either WT or HEL* proteins and infected with MERS-CoV at an MOI of 1 for 18 and 24 hpi. MOV10 and N protein accumulation in cytoplasmic extracts was detected by Western blotting. GAPDH was used as a loading control. Numbers under the blots indicate the estimated levels of N protein, normalized to GAPDH levels and relative to pcDNA-transfected cells at 18 hpi. (C) Virus titers were measured at the indicated time points, from infected MOV10-KO cells previously transfected with an empty plasmid (gray) or plasmids expressing either WT (green) or HEL* (purple) proteins. The values are means from three independent infections; error bars represent SD. *, *P* < 0.05. (D) MOV10-KO cells were transfected with plasmids expressing either WT or HEL* proteins and then infected with MERS-CoV at an MOI of 0.1. At 20 hpi, cell lysates were obtained and immunoprecipitated with anti-MOV10 antibody. MOV-10 and MERS-CoV N protein were detected by Western blotting.

MOV10 is an RNA helicase that contains an N-terminal CH-rich domain and a C-terminal helicase domain containing seven helicase motifs (Fig. 7A). To address whether helicase activity of MOV10 is involved in its antiviral function, a MOV10 helicase-deficient mutant (HEL*) was generated (Fig. 7A), in which essential amino acids in the conserved helicase motifs I and II were replaced (K530A, D645N) (53). Wild-type MOV10 (WT) and the HEL* mutant were overexpressed in MOV10-KO cells, to avoid the effects of endogenous MOV10 protein, and cells were subsequently infected with MERS-CoV at a multiplicity of infection (MOI) of 1. As expected, the expression of wild-type MOV10 decreased N protein accumulation levels (Fig. 7B) and viral titers (Fig. 7C), confirming that the viral phenotype observed in MOV10-KO cells (Fig. 6) was due to the absence of MOV10. In contrast, no differences in N protein accumulation or viral titers were observed in cells expressing the HEL* mutant (Fig. 7B and C), indicating that MOV10 helicase activity was required for antiviral activity. Interestingly, the MOV10 HEL* mutant maintained the interaction with MERS-CoV N protein (Fig. 7D), suggesting that MOV10 interaction with MERS-CoV N protein is not sufficient for antiviral activity.

### MOV10 interaction with other human CoV N proteins.

**FIG 8:**
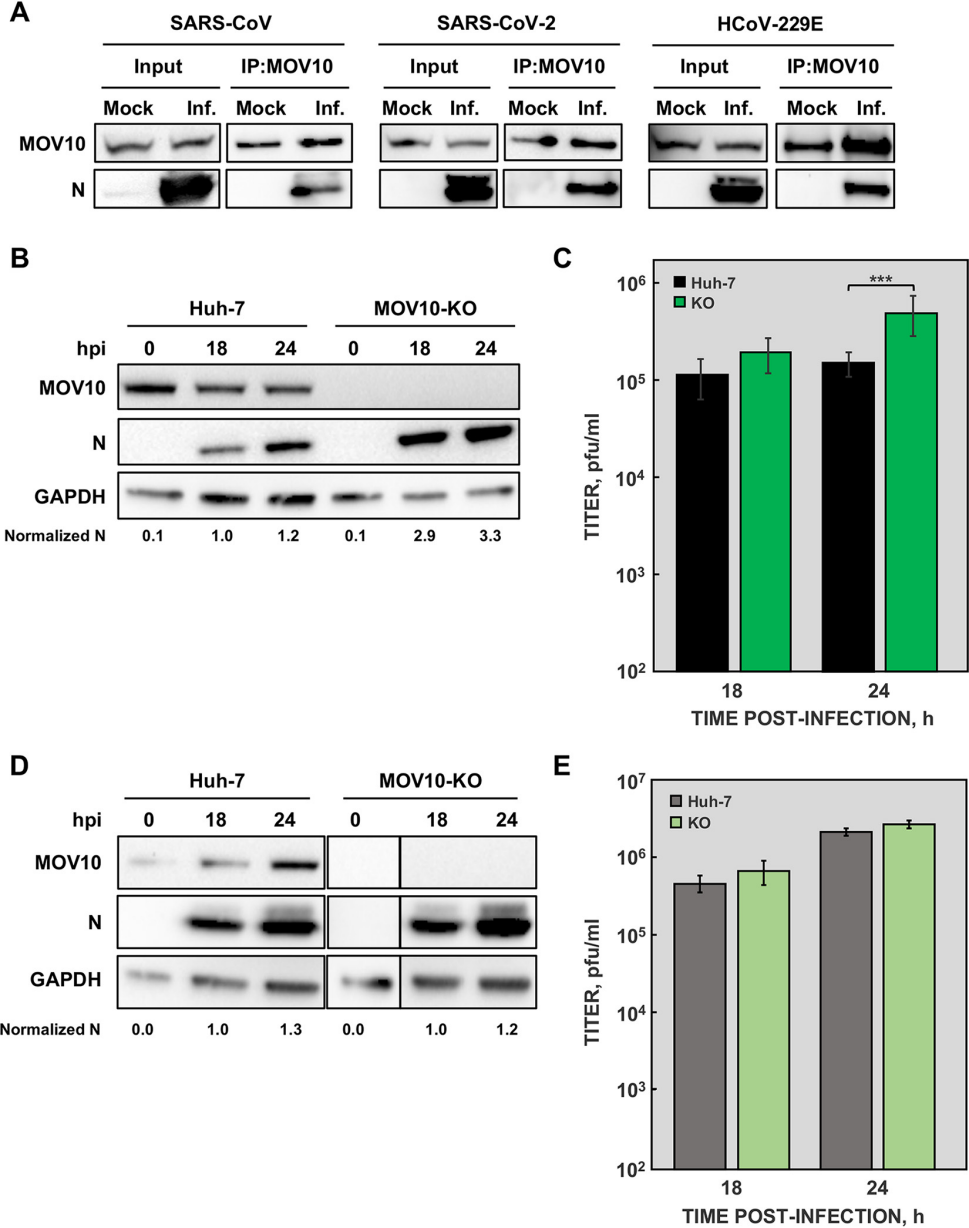
MOV10 interaction with N proteins from other human CoVs. (A) In all cases, cells were mock infected or infected with the corresponding CoV at an MOI of 0.1: DBT-ACE2 cells infected with SARS-CoV (left), Calu3 2B4 cells infected with SARS-CoV-2 (middle), and Huh-7 cells infected with HCoV-229E (right). At 20 hpi, cell lysates were obtained and immunoprecipitated with anti-MOV10 antibody. MOV10 and N proteins were then detected by Western blotting using specific antibodies. Huh-7 and MOV10-KO cells were infected with SARS-CoV-2, and both N protein accumulation (B) and viral titers (C) were analyzed at the indicated time points. Huh-7 and MOV10-KO cells were infected with HCoV-229E, and both N protein accumulation (D) and viral titers (E) were analyzed at the indicated time points. GAPDH was used as a cytoplasmic marker in all cases. Numbers under the blots indicate the estimated levels of N protein, normalized by GAPDH levels and relative to Huh-7 cells at 18 hpi. The values are means from three independent infections; error bars represent SD. ***, *P* < 0.001.

Immunoprecipitation of endogenous MOV10 also pulled down N proteins from other human-pathogenic CoVs, such as SARS-CoV and the recently emerged SARS-CoV-2 (Fig. 8A). MOV10-N protein interaction was also detected during HCoV-229E infection (Fig. 8A), indicating that MOV10-N binding is a conserved feature of CoVs.

As our previous observations indicated that MOV10-N interaction is not sufficient for MOV10 antiviral activity (Fig. 7D), we analyzed MOV10 antiviral activity in HCoV-229E and SARS-CoV-2 infection. Huh-7 cells are susceptible to infection with both SARS-CoV-2 and HCoV-229E (54), facilitating analysis of the role of MOV10 by comparing the effect of infection of native Huh-7 or Huh-7-MOV10-KO cells. In agreement with the data obtained for MERS-CoV, SARS-CoV-2 N protein accumulation was significantly increased in Huh-7-MOV10-KO cells (Fig. 8B). Moreover, a 4-fold increase in SARS-CoV-2 titers was observed in Huh-7-MOV10-KO cells compared with Huh-7 cells (Fig. 8C). In contrast, no significant difference was detected both in N protein accumulation (Fig. 8D) and virus titers (Fig. 8E) in Huh-7 cells infected with HCoV-229E in the presence or the absence of MOV10. Similar results were obtained when growth kinetics of SARS-CoV-2 and HCoV-229E were analyzed in the presence or absence of MOV10 (Fig. S3B and C). Together, these results suggested that antiviral activity of MOV10 may occur only after infection with highly pathogenic CoVs.

## DISCUSSION

A conserved interaction between human CoV N protein and cellular helicase MOV10 is described here. Moreover, functional analysis during MERS-CoV infection indicated that MOV10 plays an antiviral role, most likely as a result of viral RNA sequestration in cytoplasmic RNA-protein granules containing other cellular proteins, such as TIAR, AGO2, and UPF1, with different antiviral activities. Interestingly, the antiviral function of MOV10 was observed only in infections by the deadly human CoVs MERS-CoV and SARS-CoV-2, not with human common cold CoV HCoV-229E.

The interactions described in this work were detected between viral and endogenous cell proteins in the context of virus infection. The interaction between MOV10 and CoV N protein during infection was not previously described. Similar interaction has been reported for other CoVs, such as infectious bronchitis virus (IBV) and SARS-CoV-2, but only outside the infection context, using overexpressed N protein (55, 56). An interaction between endogenous MOV10 and the SG component TIAR was also identified, even in mock-infected cells. Although MOV10 was identified as part of the SG proteome (31), the interaction between MOV10 and TIAR has not been previously reported. Proteomics approaches have also identified MOV10 in P bodies, together with AGO2, UPF1, and Dcp1a (30). The endogenous MOV10-AGO2 and AGO2-UPF1 interactions described previously (48, 49) were identified after protein overexpression (29, 53, 57) and reinforce the biological significance of MOV10 protein interactions with viral and cellular proteins described in this work.

Our data indicated that the interaction between MERS-CoV N protein and other cellular proteins, such as TIAR, AGO2, and UPF1, was mainly mediated by MOV10. CoVs N protein location in cytoplasmic RNP granules was previously described only for SARS-CoV N protein that was overexpressed and in cell stress situations (58). Nevertheless, CoV N protein interaction with cellular proteins that are components of these structures has been reported (55, 59). There are two possible scenarios compatible with the obtained results: MOV10-N interaction is related to MOV10 antiviral function or, alternatively, N protein interacts with MOV10 to counteract its antiviral activity. MOV10-N interaction was detected in all situations analyzed, independently of MOV10 antiviral activity. In addition, it has been proposed that CoV N protein may subvert SGs or may counteract the NMD pathway to facilitate viral replication (60, 61). Additional experimental evidence is required to determine whether CoV N protein was present in the RNP granules as a consequence of viral RNA sequestration or whether it has a more active role.

MOV10-N interaction was dependent on RNA. Similarly, it has been described that MOV10-AGO2 and MOV10-UPF1 interactions were also RNA dependent (48, 53). MOV10 binds to RNA structures known as G quadruplexes, and this binding modulates MOV10 interactions with other cellular proteins and their functions (52, 62). Interestingly, G quadruplexes have been found in all CoV genomes analyzed, and even some of these RNA structures are conserved (63). In addition, during the review of this work, evidence identifying MOV10 as a protein binding to SARS-CoV-2 genomic RNA, specifically to both the 5′ and 3′ untranslated regions (UTRs), was published (50, 51). Therefore, it would be interesting to explore whether MOV10-N interaction is mediated by RNA structures present in the CoV gRNA.

MOV10 antiviral activity during MERS-CoV infection was dependent on its helicase activity. This was in contrast to the absence of a MOV10 helicase activity requirement for antiviral activity during HIV-1, influenza virus, HCV, or bunyavirus infections (40, 43, 44, 64, 65), strongly suggesting that MOV10 can exert its antiviral function by different mechanisms in different RNA viruses. Interestingly, MERS-CoV N protein was present in MOV10 complexes formed by the MOV10 mutant without helicase activity, suggesting that CoV N protein presence in MOV10 RNPs is not enough to promote its antiviral activity. In line with this observation, N protein was present in MOV10 complexes formed after infection with all human CoVs that were tested, but antiviral activity was found against only MERS-CoV and SARS-CoV-2, not HCoV-229E. The cell lines used for the analysis were identical in the infections by the three CoVs, excluding a possible effect due to the cell type. It is possible that the components of the MOV10 RNPs were different between the wild type and the helicase mutant, or between deadly human CoVs and HCoV-229E. Preliminary data indicated that the interaction between TIAR or UPF1 and the MOV10 helicase mutant was reduced (data not shown), although this preliminary observation should be further explored.

Although the MOV10 gene is an ISG (35), no differences in MOV10 expression levels were detected during MERS-CoV infection. Possibly, this was due to the absence of IFN induction in MERS-CoV-infected Huh-7 cells, as previously described (13). In fact, transfection of Huh-7 cells with poly(I-C) increased MOV10 mRNA accumulation (data not shown), confirming that the MOV10 gene is an ISG. In addition, a role for MOV10 in innate immune response modulation during virus infection was previously described (36, 42). This observation, together with the differences observed between MERS-CoV or SARS-CoV-2 and HCoV-229E virulence, opens the possibility of a role for MOV10 in CoV pathogenesis. Preliminary data on RNA IP of cellular mRNAs showed that certain host cell mRNAs were not pulled down together with MOV10, whereas other mRNAs were pulled down in both noninfected and infected cells, indicating that they were also present in the MOV10 complexes. These data led us to propose that the RNA composition of MOV10 cytoplasmic granules may be different in infected and noninfected cells. Moreover, similarly to the putative differences in MOV10 RNPs protein composition, different mRNA composition of these cytoplasmic granules during pathogenic versus mild CoV infections may affect the overall cell innate immune response. This issue will be further explored.

Together, the findings in this work showed that there is a complex network of viral and cellular RNA-protein interactions that influence the host cell antiviral response to CoVs and, most likely, the pathogenesis caused by these emergent human viruses. Moreover, by increasing our knowledge of molecular mechanisms, studies of RNA and protein components of the RNPs may help to identify novel therapeutic targets.

## MATERIALS AND METHODS

### Cell lines and viruses.

Monkey Vero cells (CCL-81) and human normal lung fibroblasts (MRC-5, CCL-171) were obtained from the American Type Culture Collection. Mouse DBT cells stably expressing the murine ACE2 receptor (DBT-ACE2), used for SARS-CoV co-IP experiments, were previously generated in our laboratory (66). Human liver-derived Huh-7 cells were kindly provided by R. Bartenschlager (University of Heidelberg, Germany). The bronchial epithelial cell line Calu-3 2B4 (67), used for SARS-CoV-2 co-IP experiments, was kindly provided by C. T. Tseng (University of Texas Medical Branch, USA). Cells were cultured in Dulbecco’s modified Eagle medium (DMEM; Lonza) supplemented with 25 mM HEPES, 10% fetal bovine serum (FBS; HyClone), 2% glutamine, and 1% nonessential amino acids (Sigma) and maintained at 37°C in a humidified atmosphere of 5% CO_2_. To maintain DBT-ACE2 cells, 1 mg/ml of G418 was added to the medium. Calu-3 2B4 cells were grown in the medium described above supplemented with 20% FBS.

Mouse-adapted MERS-CoV and SARS-CoV-MA15 were rescued from the corresponding infectious cDNAs (16, 68). SARS-CoV-2 was isolated from a clinical sample from a hospital in Madrid (J. M. Honrubia and L. Enjuanes, unpublished results). HCoV-229E was kindly provided by V. Thiel (Institute of Virology and Immunology, Switzerland). MERS-CoV virus growth and titration were performed as previously described (9). All experiments with infectious MERS-CoV, SARS-CoV, and SARS-CoV-2 were performed in biosafety level 3 (BSL-3) facilities at CNB-CSIC according to the guidelines set forth by the institution.

### Plasmid constructs.

The plasmid for human MOV10 expression was generated by cloning the *MOV10* cDNA sequence (GenBank accession number NM_020963) into pcDNA3.1 vector. To that end, the *MOV 10* sequence was amplified with the oligonucleotides 5′-MOV (5′-GCACG*CTCGAG*ATGCCCAGTAAGTTCAGCTGCCGGCAG-3′; XhoI restriction site in italics) and 3′-MOV (5′-GGCTGG*GGATCC*TCAGAGCTCATTCCTCCACTCTGGCTCC-3′; BamHI site in italics), using as a template plasmid pbcbe-flag-mov10, kindly provided by G. Peters (London Research Institute, UK) (32). The PCR product was digested with XhoI and BamHI and cloned into the same sites of pcDNA3.1. To generate the mutant lacking helicase activity (HEL*), a DNA fragment containing nucleotides 1724 to 3066 of *MOV10* gene and two point mutations (K530A and D645N) was chemically synthesized and purchased from GeneArt (Thermo Fisher). The mutant DNA was digested with BstEII and BsrGI and cloned into the same sites of pcDNA-MOV10. All the cloning steps were checked by sequencing.

### Transfection of cells.

For wild-type and mutant MOV10 protein expression, cells were reverse transfected with MOV10 expression plasmids using Lipofectamine 2000 (Invitrogen) according to the instructions of the manufacturer. After 24 h, G418 was added at a final concentration of 1 μg/μl, and selection was maintained for 2 days before infection and subsequent analyses. For small interfering RNA (siRNA) knockdown of MOV10, Huh-7 cells (1.2 × 10^6^ cells per well in an M6 plate) were transfected with 100 nM MOV10 siRNA (si8922, Ambion) or negative-control siRNA (Ambion) using TransIT-X2 (Mirus Bio) transfection reagent according to the manufacturer’s specifications. After 48 h, cells were retransfected with 25 nM siRNAs and incubated for 24 h. Two additional siRNAs specific for MOV10 were tested (si8923 and si8924) with very limited MOV10 silencing in Huh-7 cells; these siRNAs were therefore not selected for subsequent experiments. Cells were then infected with MERS-CoV for further analysis.

### Generation of the Huh7_*MOV10*_KO (MOV10-KO) cell line.

The MOV10-KO cell line was generated in Huh-7 cells using the CRISPR/Cas9 system. Guide RNA of *MOV10* was designed online using the Breaking-Cas tool (69). Critical exons of the *MOV10* gene were targeted using the guide RNA 5′-GGTTCTTCAGACTCGACCGC**TGG**-3′´ (protospacer-adjacent motif [PAM] region in boldface). A pair of oligonucleotides comprising the guide RNA sequence flanked by BbsI restriction sites was designed. Oligonucleotides were annealed, digested with BbsI, and cloned into the same sites of the CRISPR plasmid pX330 (kindly provided by P. A. Mateos-Gomez, University of Alcala de Henares, Spain). Cells were transfected with 2 μg of pX330-guide RNA construct using Lipofectamine 2000 according to the manufacturer’s protocol. At 24 h posttransfection (hpt), puromycin (2 μg/ml) selection was introduced for an additional 48 h. Subsequently, the cells were cloned several times by limiting dilution, and three independent clones were then amplified for later use. Genotype and phenotype of MOV10-KO cell line were confirmed by sequencing and Western blotting, respectively. Moreover, the absence of four predicted off-targets in the MOV10-KO cell line was determined by PCR and sequencing.

### Analysis of RNA by RT-qPCR.

Total intracellular RNA was extracted with an RNeasy minikit (Qiagen) according to the manufacturer’s instructions. Total cDNA was synthesized with random hexamers from 100 ng of total RNA as a template using a high-capacity cDNA reverse transcription kit (Applied Biosystems), following the manufacturer’s recommendations. Cellular gene expression was analyzed using a human *MOV10*-specific TaqMan gene expression assay (Hs00918631_m1) and the hydroxymethylbilane synthase (HMBS) gene (Hs00609297_m1) as a reference housekeeping gene. MERS-CoV genomic RNA (gRNA) and subgenomic RNA (sgmRNA) were evaluated using custom TaqMan assays previously described (9). Data were acquired with a 7500 real-time PCR system (Applied Biosystems) and analyzed with 7500 software v2.0.6. Relative quantifications were performed using the 2^−ΔΔ^*^CT^* method (70). All experiments and data analysis were MIQE (Minimum Information for Publication of Quantitative Real-Time PCR Experiments) compliant (71).

### Nuclear and cytoplasmic fractionation.

Nuclear and cytoplasmic fractions from Huh-7 cells were prepared following a protocol adapted from reference 72. Briefly, cell monolayers were washed three times with ice-cold phosphate-buffered saline (PBS), collected, and resuspended in lysis buffer (0.1% IGEPAL CA-630 [Sigma] in PBS). Extracts were centrifuged at 10,000 × *g* for 1 min to recover the cytoplasmic fraction in the supernatant and the nuclei in the pellet. The cytoplasmic fraction was mixed with Laemmli sample buffer, 1:1. The pellet was resuspended in lysis buffer and centrifuged at 10,000 × *g* for 1 min. The supernatant was removed, and the nucleus pellet was mixed with Laemmli sample buffer, 1:1. The purity of the fractions was checked using antibodies specific for cytoplasmic (anti-GAPDH [1:3,000; Cell Signaling]) or nuclear (anti-His3 [1:10,000; Cell Signaling]) proteins.

### Protein analysis by Western blotting.

Whole-cell protein extracts were prepared in loading buffer (0.1 M Tris-HCl, pH 6.8; 20% glycerol; 4% [wt/vol] SDS; 0.2% bromophenol blue; and 0.05% β-mercaptoethanol). Proteins from whole-cell or cytoplasmic extracts were analyzed by denaturing electrophoresis in NuPAGE 4 to 12% bis-Tris gels with 3-morpholinopropane-1-sulfonic acid (MOPS) SDS running buffer (Invitrogen). Proteins were transferred to a polyvinylidene difluoride (PVDF) membrane (Bio-Rad) and incubated with the primary antibodies rabbit anti-MOV10 [ab80613] (1:2,000; Abcam), rabbit anti-MERS-CoV N (1:2,000; Sino Biological), rabbit anti-SARS-CoV-2 N (1:2,000; Sino Biological), mouse anti-HCoV-229E N (1:1,000) (73), goat anti-TIAR (1:1,000; Santa Cruz Biotechnology), goat anti-UPF1 (1:1,000; Bethyl), rat anti-AGO2 (1:2,000; Sigma), goat anti-ATP5B (1:1,000; Santa Cruz Biotechnology), rabbit anti-GAPDH (1:3,000; Cell Signaling), rabbit anti-histone H3 (1:10,000; Cell Signaling), or rabbit anti-β-actin (1:5,000; Cell Signaling). Species-specific secondary horseradish peroxidase (HRP)-conjugated antibodies were used (1:10,000, Sigma). The immune complexes were detected using Clarity Western enhanced chemiluminescence (ECL) blotting substrate (Bio-Rad) and a ChemiDoc XRS^+^ system (Bio-Rad), according to the manufacturer’s instructions. Protein amounts were estimated by densitometric analysis using ImageLab 6.1 software (Bio-Rad). At least three different experiments and appropriate gel exposures were used in all cases with similar results. In addition, different exposures of the same experiment were analyzed to ensure that data were obtained from films within the linear range.

### Immunofluorescence analysis.

Huh-7 or MRC-5 cells were grown on 13-mm glass coverslips and infected with MERS-CoV at a multiplicity of infection (MOI) of 0.1. At 20 h postinfection (hpi), cells were fixed and the virus was inactivated by incubation with 4% paraformaldehyde in PBS for 40 min at room temperature. Cells were permeabilized with ice-cold methanol for 10 min and blocked with 10% FBS in PBS for 1 h at room temperature. Primary antibodies were diluted in PBS with 5% FBS as follows: mouse anti-MOV10 (15C1B8), 1:500 (Bethyl); rabbit anti-MERS-CoV N protein, 1:500 (Sino Biological); and rabbit anti-HCoV-229E-nsp8, 1:400 (kindly provided by John Ziebuhr, Giessen University, Germany). Cells were then washed five times for 5 min each time with PBS and incubated for 30 min at room temperature with species-specific secondary antibodies conjugated to Alexa Fluor 488 or Alexa Fluor 546, diluted 1:500 in PBS with 5% FBS. Cell nuclei were stained with Hoechst 33342 (1:200; Sigma). Finally, coverslips were mounted in Prolong Gold antifade reagent (Invitrogen). Confocal microscopy was performed using a Leica SP8 laser scanning microscope, and images were collected and processed with LAS AF software (Leica, Wetzlar, Germany). To calculate Pearson´s correlation coefficients, three areas per image of 120 by 120 pixels with high MOV10 accumulation were analyzed. At least 20 cells in two different experiments were analyzed.

### Co-IP.

Huh-7 and MOV10-KO cell extracts were collected at 20 hpi and lysed in IP buffer (50 mM Tris [pH 8.0], 1% NP-40, 300 mM NaCl, and 5% glycerol) with protease inhibitor cocktail (Roche). Fifty microliters of protein G Dynabeads (Invitrogen) was prepared as recommended by the manufacturer, and corresponding antibodies diluted in IP buffer were added. Following 2 h of incubation at 4°C with rotation, the beads were washed three times with wash buffer (10% Surfact-Amps X-100 in PBS [1×; pH 7.2]). Proteins were eluted by the addition of SDS loading buffer directly to the washed beads. Protein samples were boiled for 10 min and analyzed by Western blotting as described above. Antibodies used in co-IP were mouse anti-MOV10 (15C1B8) (1:20; Bethyl), goat anti-TIAR (1:50; Santa Cruz Biotechnology), rabbit anti-UPF1 (1:50; Cell Signaling), rat anti-AGO2 (1:50; Sigma), and goat anti-ATP5B (1:20; Santa Cruz Biotechnology).

### RNA IP.

Huh-7 cells were mock infected or infected with MERS-CoV at a MOI of 0.1. At 20 hpi, extracts were recovered in IP buffer with protease inhibitor cocktails (Roche) and 1.6 U/μl of RNasin RNase inhibitor (Promega), incubated at 4°C for 10 min, and centrifuged at 3,000 × *g* for 2 min at 4°C. The supernatant was collected and precleared with protein G Dynabeads before IP, following the manufacturer’s instructions. Rabbit anti-MOV10 and rabbit anti-green fluorescent protein (anti-GFP; Boehringer Mannheim) antibodies were first bound to protein G Dynabeads (10 μg/μl) diluted in IP buffer and incubated with rotation for 2 h at 4°C, and then cell extracts were added to magnetic beads. Immunoprecipitated RNA-protein complexes were eluted according to the manufacturer’s instructions. RNA was isolated with an RNeasy kit (Qiagen) and subjected to RT-qPCR for the detection of MOV10- or GFP-associated viral RNAs as described above.

### Statistical analysis.

Two-tailed, unpaired Student’s *t* tests were used to analyze the difference in mean values between groups. All results are expressed as means and standard deviations (SD). *P* values of <0.05 were considered significant.
