# Supplementary figures and images for: MOV10 Helicase Interacts with Coronavirus Nucleocapsid Protein and Has Antiviral Activity
Source: mBio. 2021 Sep 14;12(5):e01316-21. doi: 10.1128/mBio.01316-21 (PMC8546642; doi:10.1128/mBio.01316-21)

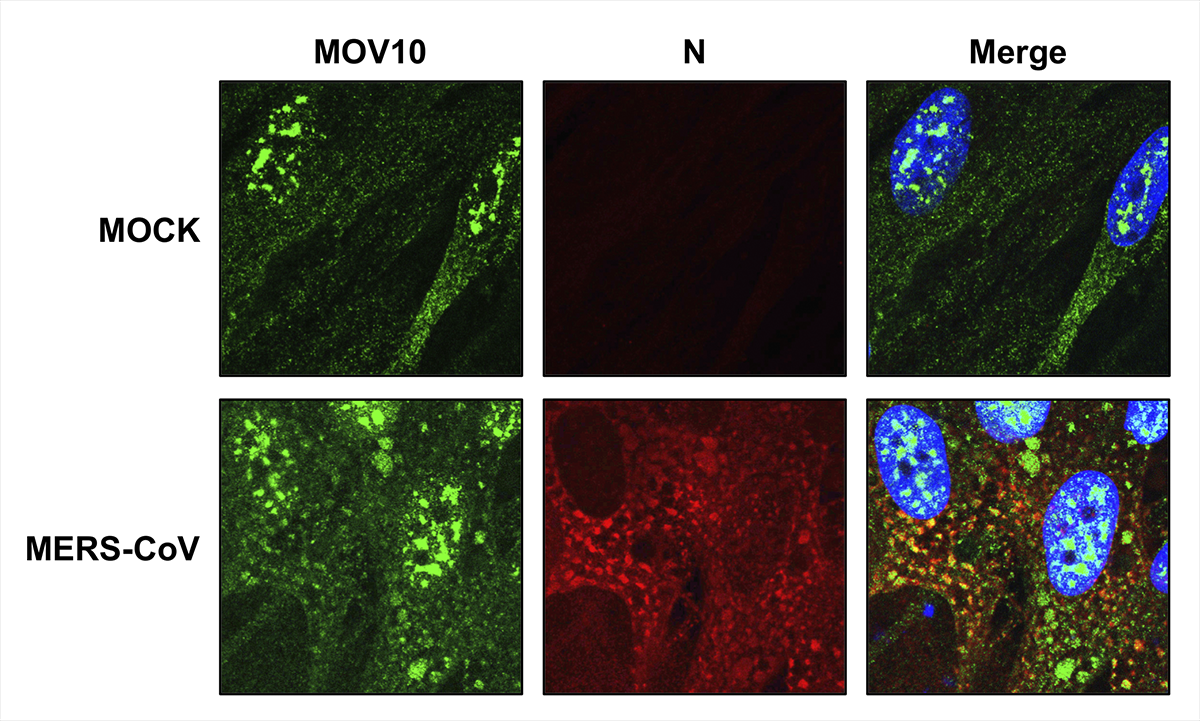

Supplement: FIG S1 [file mbio.01316-21-sf001.tif]

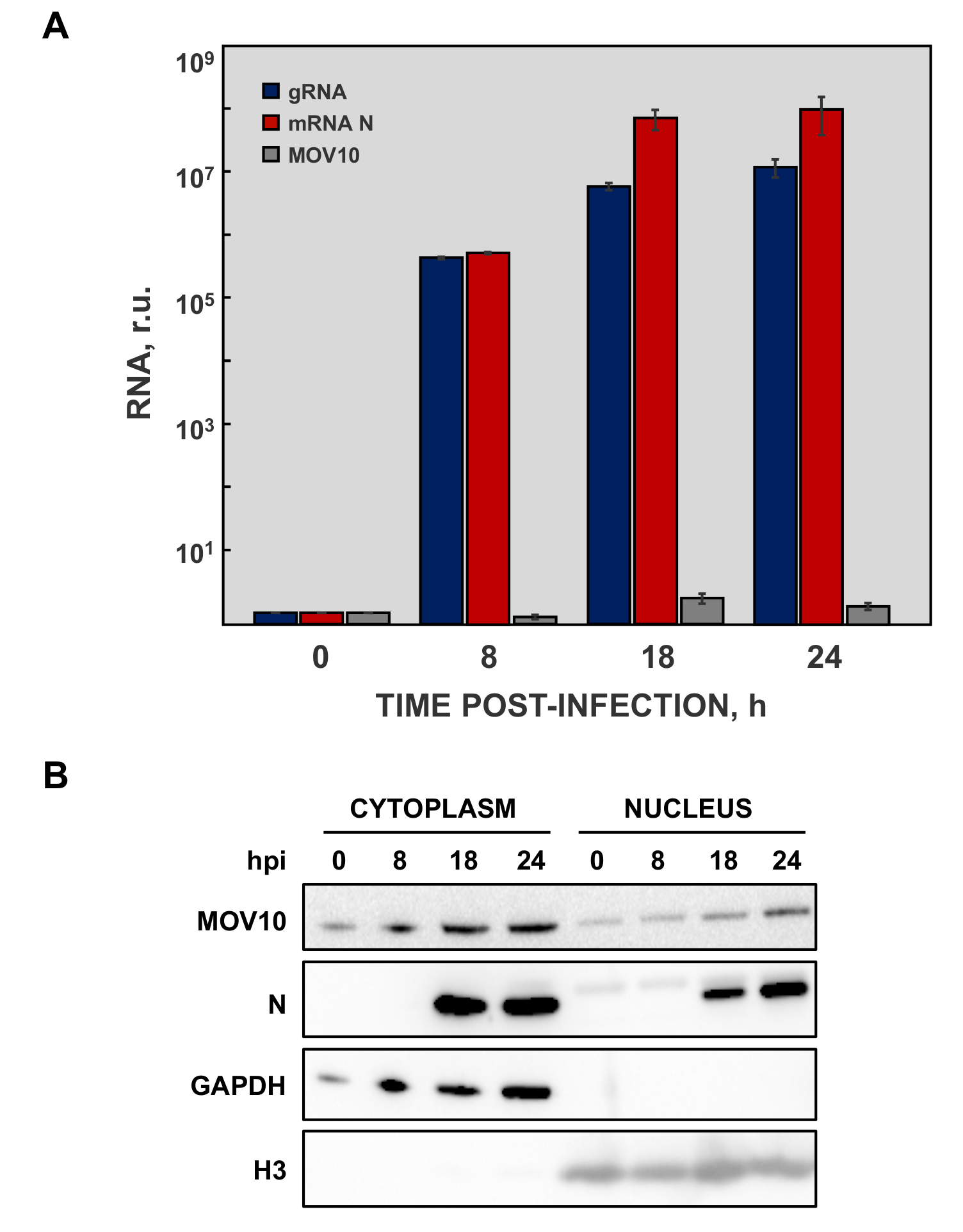

Supplement: FIG S2 [file mbio.01316-21-sf002.tif]

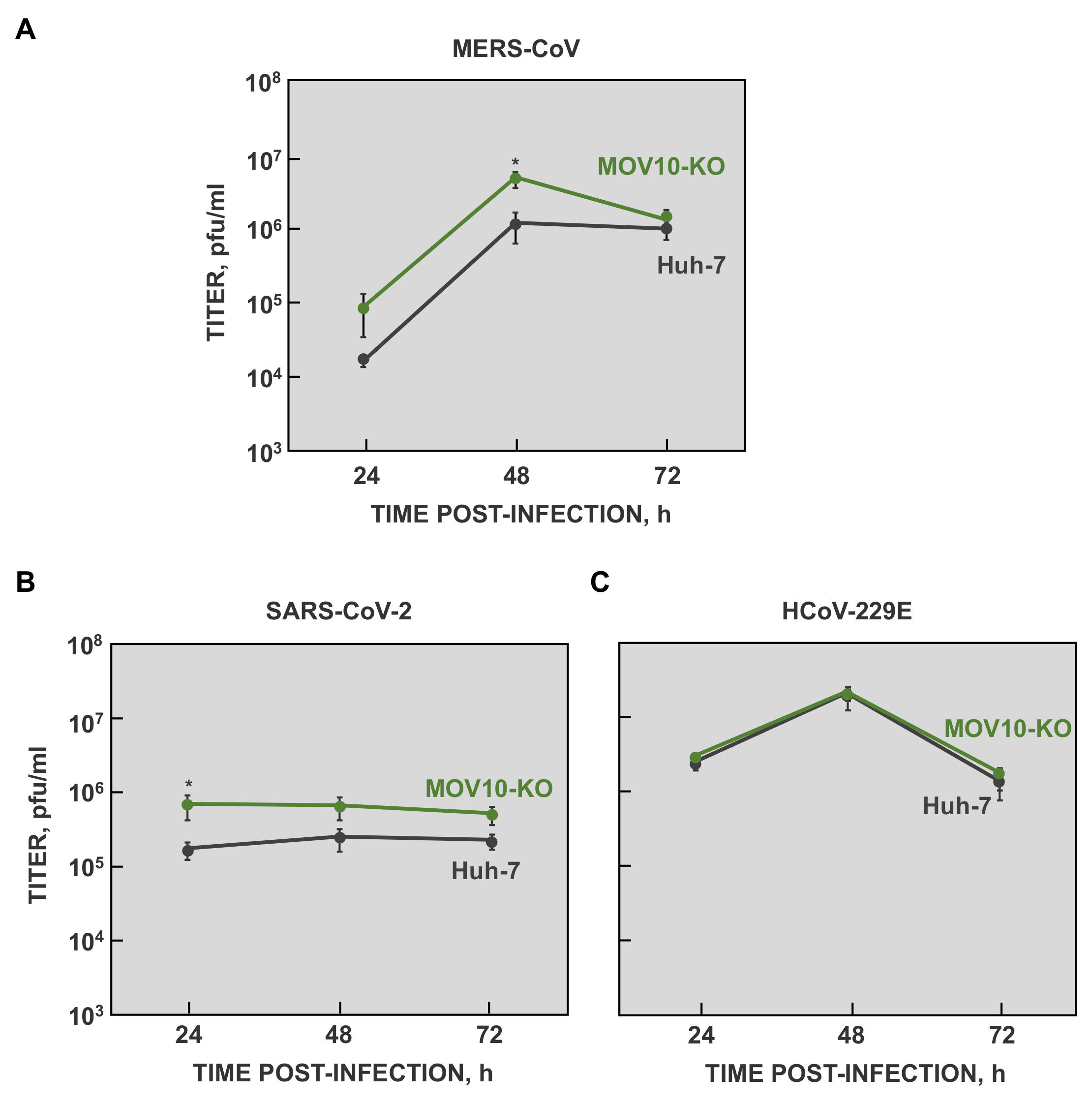

Supplement: FIG S3 [file mbio.01316-21-sf003.tif]
